# Smart Glasses for Caring Situations in Complex Care Environments: Scoping Review

**DOI:** 10.2196/16055

**Published:** 2020-04-20

**Authors:** Charlotte Romare, Lisa Skär

**Affiliations:** 1 Region Blekinge Karlskrona Sweden; 2 Department of Health Blekinge Institute of Technology Karlskrona Sweden

**Keywords:** anesthesia department, critical care, intensive care units, scoping review, smart glasses

## Abstract

**Background:**

Anesthesia departments and intensive care units represent two advanced, high-tech, and complex care environments. Health care in those environments involves different types of technology to provide safe, high-quality care. Smart glasses have previously been used in different health care settings and have been suggested to assist health care professionals in numerous areas. However, smart glasses in the complex contexts of anesthesia care and intensive care are new and innovative. An overview of existing research related to these contexts is needed before implementing smart glasses into complex care environments.

**Objective:**

The aim of this study was to highlight potential benefits and limitations with health care professionals' use of smart glasses in situations occurring in complex care environments.

**Methods:**

A scoping review with six steps was conducted to fulfill the objective. Database searches were conducted in PubMed and Scopus; original articles about health care professionals’ use of smart glasses in complex care environments and/or situations occurring in those environments were included. The searches yielded a total of 20 articles that were included in the review.

**Results:**

Three categories were created during the qualitative content analysis: (1) smart glasses as a versatile tool that offers opportunities and challenges, (2) smart glasses entail positive and negative impacts on health care professionals, and (3) smart glasses' quality of use provides facilities and leaves room for improvement. Smart glasses were found to be both a helpful tool and a hindrance in caring situations that might occur in complex care environments. This review provides an increased understanding about different situations where smart glasses might be used by health care professionals in clinical practice in anesthesia care and intensive care; however, research about smart glasses in clinical complex care environments is limited.

**Conclusions:**

Thoughtful implementation and improved hardware are needed to meet health care professionals’ needs. New technology brings challenges; more research is required to elucidate how smart glasses affect patient safety, health care professionals, and quality of care in complex care environments.

## Introduction

### Complex Care Environments

Improvements in medical skills and technology have made health care increasingly complex [1]. Anesthesia departments and intensive care units (ICUs) represent two advanced, high-tech, and complex care environments [2,3]. In the anesthesia department, patients undergo planned or acute surgeries, treatments, or examinations. The patients are often under sedation or anesthesia, which affects vital organ functions. Specialized health care professionals are responsible for maintaining the patient’s ventilation and handling changes in the homeostatic balance caused by sedation or anesthesia. Advanced technology, such as ventilators, physiological monitoring, and the anesthesia station, make this possible [2]. The most critically ill patients are admitted to the ICU. These patients can have failure in one or more vital organ systems, such as the cardiovascular, respiratory, or renal system. Numerous examinations and treatments are performed and used, such as mechanical ventilation, bronchoscopy, dialysis, and multiple potent drugs [3]. In both ICUs and anesthesia departments, changes in the patient’s condition can occur rapidly and may demand an immediate response from health care professionals to save the patient’s life, hence, close surveillance is vital. Health care in these complex care environments is based on well-trained and dedicated health care professionals, teamwork, and the use of technology to provide high-quality care and ensure patient safety [2,3]. Caring situations in complex care environments include, for example, advanced medical, technological, and caring components and the surrounding specific environment. In this study, we use the expression *complex care environment* to describe all these aspects in the contexts above.

### Patient Safety

Patients being cared for in complex care environments are in a vulnerable state, due to their conditions and the treatments they need. According to the World Health Organization, patient safety work aims to prevent avoidable patient harm and provide a safe health care environment. They also state that delivering safe complex care is a challenge [4]. The use of advanced technology, such as ventilators and physiological monitoring, is a prerequisite for care in anesthesia departments and ICUs. Technology is known to increase patient safety and to enhance patient care [5], but technology also imposes risks. In 2019, the Emergency Care Research Institute (ECRI) included both ventilators and physiological monitoring on their annual top-10 list of health technology hazards [6]. This imposes continuous work for patient safety in complex care environments. Patient safety work is not only related to the use of technology. In complex situations, several factors interact; patient safety work is also related to other aspects, for example, working conditions and routines [4,7]. Health care professionals in complex care environments incorporate several factors into their surveillance during patient care in order to provide safe care [8]. Through close surveillance, health care professionals can support both the physical and emotional needs of the patient, to protect the patent from suffering and harm. This promotes a patient-safe way of working [9], as do proper implementation and use of new technology [7]. It is also important for new technology to add value to patient care and to bring desired consequences [10].

### Smart Glasses

Smart glasses are a product suggested to aid health care professionals in numerous areas, such as surgery, accessing electronic health records, remote instructions, and education [11-13]. They are a computing device worn as a pair of glasses, which presents information within the user’s field of view through a prism. Smart glasses are a platform for apps and can display text and images, use a camera, and communicate via Bluetooth and Wi-Fi. The user interacts with the smart glasses through physical input or voice commands [14]. Smart glasses can send and receive information online, or through local area networks, and the information can be displayed in the prism. Smart glasses can also be used to communicate by voice or video and to capture pictures or video. The uses for smart glasses depend on the apps in the device; tailored apps provide the possibility for multiple purposes. The most well-known brand of smart glasses today is Google Glass, which was introduced to the market in 2013.

### Smart Glasses in Complex Care Environments

According to our literature search, a few reviews have been published about smart glasses in surgical and nonsurgical settings. Different areas of use are described, such as to provide visualization during laparoscopy, to broadcast live surgery to medical students, to take pictures and record videos to facilitate medical documentation, to record encounters with patients, and to use as a navigational tool to maintain attention to the operative field [11-13,15-19]. The idea of head-mounted and hands-free equipment as an aid in anesthesia departments is not new [20]; health care professionals have shown interest in, and have seen the potential for, smart glasses in intensive care [21]. Since there is a growing interest in smart glasses and since technology might have an effect on patient safety, it is important to conduct a scoping review on smart glasses used by health care professionals in complex care environments in order to identify the knowledge and experiences in this field. In our study we use the term *health care professionals* to describe physicians with different levels of experience and training, registered nurses, specialized nurses, and other professionals working closely with patients, such as assistants. To our knowledge, only a few studies have been performed within our area of interest [22,23]. This indicates that the use of smart glasses in complex care environments is a new and evolving area, making it even more important to investigate. This field is novel, innovative, and has been found to have potential to improve both patient care and patient safety in other health care settings [16,19]. The aim of this study was, therefore, to highlight potential benefits and limitations with health care professionals’ use of smart glasses in situations occurring in complex care environments.

## Methods

### Design

A scoping review was chosen as the methodology of this study since it addresses broad research questions and is advocated for new areas [24-26]. This review followed all six stages suggested by Arksey and O’Malley [25] and the methodological development by Levac et al [27]. Results are reported according to the PRISMA (Preferred Reporting Items for Systematic Reviews and Meta-Analyses) extension for scoping reviews (PRISMA-ScR) in order to increase methodological transparency [26].

### Data Search and Selection

Step 1 was to identify the research question; a scoping review approach has been suggested [25,27] and our path toward the final aim was described in the Introduction. Step 2 was to identify relevant articles. Before we began the database searches, we consulted experienced librarians who assisted in choosing the most appropriate databases and search terms as suggested by Arksey and O’Malley [25]. PubMed and Scopus were chosen to cover research within both health care and engineering. Search terms were also discussed among the authors and with other researchers within the fields of nursing, medicine, and engineering. New search terms were added several times during the process. The final search terms are presented in Multimedia Appendix 1. As stated initially, only two articles were identified during our initial searches [22,23], which made us broaden our searches to include vital signs monitoring, alarm management, and patient safety, since these are important aspects of care in complex care environments. This resulted in one additional article from a clinical setting [28] and two from simulated settings [29,30]. We also found articles about isolated events occurring in complex care environments, such as electrocardiogram (ECG) reading and cardiopulmonary resuscitation (CPR). We did not actively search for specific isolated events using those words as search terms in the database searches, but we did include articles found during our searches. The database searches are presented in Multimedia Appendix 2.

Additional articles were identified using reference lists and research networks (ie, ResearchGate and Academia). The deadline for searching databases was set to April 2018, and the deadline for searching other sources was set to December 2018. Step 3 was the study selection of the scoping process [25,27]. The inclusion and exclusion criteria are presented in Textbox 1. We made no limitations on the publication date, since smart glasses are a new product.

**Figure 1:**
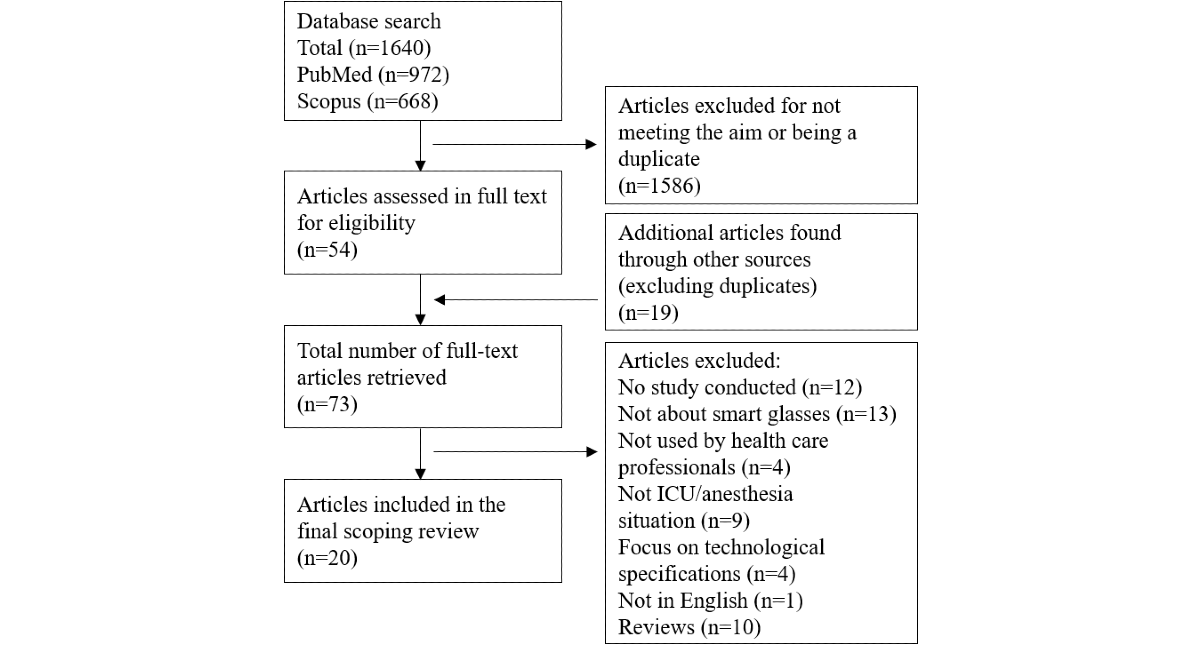
Flowchart of search.

Titles in the search results list were screened first, followed by the abstracts, if needed, in order to identify relevant articles. The full-text articles were obtained and read if they seemed eligible for this review. Screening was performed by the first author (CR) and discussed among the authors. A flowchart of the search process, similar to a PRISMA 2009 flow diagram [31], is presented in Figure 1.

**Inclusion and exclusion criteria for publications.**

**Inclusion criteria**

- Smart glasses used in complex care environment
- Smart glasses used in situation occurring in complex care environment
- Smart glasses used by health care professionals
- Written in English

**Exclusion criteria**

- Smart glasses used by students
- Smart glasses used by patients
- Review article

### Data Summary and Analysis

Step 4 involved charting the data to gain an overview. This charting is presented in Multimedia Appendix 3. During step 5, the results were collated, summarized, and reported [25]. This can be a challenging process and it is recommended to divide step 5 into three parts: analysis, reporting results, and considering the overall implications of the results. A qualitative content analysis is recommended [27], hence, we chose to follow Polit and Beck’s [24] description of this process. Meaning units meeting the aim of this study were marked in the included articles and condensed while still retaining the core content. Notes about context were added to the condensed units. The condensed units were then continuously numbered, labelled with a code, sorted into subcategories, and then sorted into categories; hence, analysis was on a manifest level [24]. Neither the analysis nor the scoping process occurred in a one-way direction but went back and forth between the steps as more knowledge was obtained. Step 6—the last step—in the scoping process was to enable practitioners and consumers to contribute to the work. The results of this scoping review have been presented to, and discussed with, engineers, a physician, registered nurses, and nurses specialized in intensive care and anesthesiology.

## Results

### Overview

The aim of this study was to highlight potential benefits and limitations of health care professionals’ use of smart glasses in situations occurring in complex care environments. A total of 20 articles [22,23,28-30,32-46] were found eligible for our scoping review and were included in the content analysis (see Multimedia Appendix 3). These included research articles (16/20, 80%), conference articles (2/20, 10%), a case report (1/20, 5%), and a correspondence (1/20, 5%). The included articles originated from the United States (12/20, 60%), European countries (7/20, 35%), and Australia (1/20, 5%) and were published in a variety of scientific journals and conference proceedings. One article was published in 2012; the rest were published between 2014 and 2018. A majority of the articles were from simulated or laboratory settings (11/20, 55%) and 1 article out of 20 (5%) was conducted in both a simulated and clinical setting; both qualitative and quantitative designs were used. During analysis, three categories were created: (1) *Smart glasses as a versatile tool that offers opportunities and challenges*, (2) *Smart glasses entail positive and negative impacts on health care professionals,* and (3) *Smart glasses’ quality of use provides facilities and leaves room for improvement*.

### Smart Glasses as a Versatile Tool That Offers Opportunities and Challenges

Smart glasses were found to be used in several situations occurring in complex care environments, including in daily practice [22,32], for vital signs monitoring [28-30], for consultation and assessment [33,34], for CPR evaluation [35,36], for documentation (ie, verbal, photo, and video) [37-40], and for viewing medical images [41-46].

Smart glasses were found to be easy to use in procedural settings [30]. Procedures were performed correctly [45,46] and with equivalent technique, both with and without smart glasses [29,35]. Increased time for completing tasks was noted when using smart glasses [37,46]. When using smart glasses for vital signs monitoring, abnormal signs were noted earlier than with traditional monitoring [29,30]; smart glasses were found suitable for this purpose [28,30] and increased awareness of vital signs [29,30]. Even though smart glasses made it easier to monitor vital signs, especially if working alone, health care professionals did not feel that smart glasses could replace the traditional monitor [30]. Presenting vital signs in smart glasses made uninterrupted monitoring possible, even when engaged in other activities [23,32] or at a remote location [23].

Smart glasses provided the possibility to share information with colleagues [22,32,37,40]. In some cases, visual media from smart glasses caused the remote consultants to change the management plan for patients. The remote consultants mostly gained confidence in the management plans and found the visual media helpful [33]. Assessing patients remotely through smart glasses showed high agreement with on-site investigators, with assessment of pupil size as the least correlating parameter [34]. Gaining expert help through smart glasses’ audio-video link during CPR was found to be helpful and reassuring. Technique and management improved, but CPR was sometimes interrupted, both because of the instructions given and by discussions with the remote expert [35]. Research found that smart glasses were eligible for educational purposes as well as for accessing patient medical records [32] and databases [22]. Furthermore, smart glasses were used to read patient barcodes for patient identification in order to increase patient safety [38]. The possibility of increasing patient safety through smart glasses was also mentioned by others [30,32].

Smart glasses could facilitate documentation, although text from voice recognition in a medical context needed improvement [22,38]; poor audio quality was seen as a contributing factor. Text was usually recognized when health care professionals talked clearly and slowly [22]. A context-specific vocabulary was suggested and the ability to review and edit the text was seen as necessary [38]. The default setting for both audio [38] and video recordings needed to be longer in order to be useful in clinical practice [22,38]. Smart glasses were found to be easy to use for video recordings [36] and provided good quality [22,39]. Video from smart glasses was rated better than video from a standard video camera. Both visibility and audibility were equivalent to that of an on-site observer. Health care professionals stated that they would be uncomfortable recording an actual event [36], and some were worried that they would be filmed unknowingly [22]. There was some discrepancy between what the user saw and what was recorded. In order to capture the right area of interest, the user had to angle the head [22,35] to an uncomfortable position [22]. Difficulties in capturing the correct area of interest were also noted when taking photos with smart glasses; this and a decrease in sharpness were the main differences between smart glasses and an ordinary digital camera [37]. Others found no difference between photographing with smart glasses and an ordinary digital camera, but they preferred to preview photos on a larger screen than in smart glasses [38]. Some aspects of complex care were seen to be best documented by a photo [39] that could easily be captured by smart glasses, hands-free and without assistance [37-39]. Since smart glasses lack the ability to zoom, health care professionals sometimes had to come closer to the photo object than they preferred [22,37].

The small size of the smart glasses’ display caused dissatisfaction when working with medical images, as did the lack of zoom [41]. Health care professionals found it difficult to notice subtle findings in medical images [22]. To improve the concept, high-quality images were requested [41] and the provided ability to zoom and pan was appreciated by users [44]. Health care professionals were not confident in their interpretations of medical images in smart glasses [41], and interpretations were less correct when performed in smart glasses than when performed traditionally [41,43,44]. When interpreting streamed ECG in smart glasses, no difference was noted from standard conditions regarding noticing different rhythms [42].

### Smart Glasses Entail Positive and Negative Impacts on Health Care Professionals

Smart glasses were described as new tools for health care professionals in the included articles. Health care professionals felt unfamiliar with smart glasses [23,36] and noted that there was a learning curve [30,44,45]. If the smart glasses’ camera was used improperly, the quality of images was affected [38]; practice [22] or training courses [40] were suggested. Health care professionals’ general impressions of smart glasses were positive [22,23,30,40] and they stated that they would like to use smart glasses again [23,29,46]. Health care professionals did not feel interrupted or disturbed by smart glasses during procedures or patient management [22,23,30,33,37,39]. No objective or subjective nervousness or anxiety were found [29], although some health care professionals did feel distracted by the smart glasses [29,35,36], which affected their performance negatively [29,35]. Increased focus on, and quality of, the task performed using smart glasses were noted as a positive aspect; however, on the negative side, it was difficult to talk to the patient and to the smart glasses at the same time [40]. When using smart glasses during procedures, health care professionals gained increased focus on the procedural field, and ergonomics improved since they did not have to turn their heads to view monitors [30,45,46]. Health care professionals did, however, spend more time looking at the smart glasses display than they did at a traditional ultrasound screen [46]. Smart glasses were found to be comfortable to wear [23,29,34,35,37,46]. Some users who wore prescription glasses found it difficult to combine these with smart glasses [23,29,35], while others did not have this issue [36]. On smart glasses where the prism was fixed to the right eye, left-handed users reported discomfort [29]. Health care professionals reported eye strain and fatigue after using smart glasses [23,36,42]. Some health care professionals did not find it problematic to use smart glasses the whole day, while others found it infeasible [40].

### Smart Glasses’ Quality of Use Provides Facilities and Leaves Room for Improvement

This category involves aspects of technical performance, navigation, and hardware. The quality of photos and videos captured by smart glasses was positively evaluated [22,33,35,37,38,43-45], although photos from an ordinary digital camera received higher ratings [37]. With adequate lighting, no photos were over- or underexposed [38]. Smart glasses had no flash, which led to decreased photo quality in low-light environments, and overexposure occurred with overhead operating lamps [22]. Furthermore, the absence of the ability to zoom affected the possibility of getting the correct area in focus for the photos [22,37]. The smart glasses display was easily seen [22,23] and the contrast improved if the background was dark. During videoconferencing, small letters were not legible [22] and the display was considered too small to provide all details on medical images, such as radiographs [43]. When communicating with others using smart glasses, the room needed to be quiet for good audibility [22].

Wi-Fi and/or Bluetooth were used for data transmission. Smart glasses were able to connect to Wi-Fi and Bluetooth without problems [22], but issues with Wi-Fi coverage were noted [23,34,38]. During data transmission, stuttering, cutoffs, and delays occurred [22,23,34,46]. Data saved in smart glasses were automatically uploaded to a cloud server when smart glasses were charged and connected to Wi-Fi. This could be avoided by connecting smart glasses to a computer prior to charging in order to transfer and delete data without uploading it to the server [22].

Smart glasses could be controlled by voice or physical input, such as using a touch pad, eyeblinks, or head movements. Controlling smart glasses through a temple touch pad was found to be easy and intuitive. In sterile environments [22] and when hands were busy or contaminated [38], hands-free handling was found useful. Voice control worked well in both silent and busy environments [37], but problems with voice control for video recordings were reported [33,38]. Features for controlling smart glasses by gestures were tested and appreciated by users [22,37,38] but did not always work well in practice, due to unintentional input [22,38].

Issues were raised about the limited battery life of smart glasses [22,29,36,46]. When recording video or using teleconferencing, the battery lasted 30-40 minutes and otherwise up to 10 hours [22]. Smart glasses were also found to produce a noticeable amount of heat [28,36,46]. When used clinically, smart glasses can be equipped with splatter eye protection [22] and were disinfected using disinfecting wipes [37] or by wiping with 70% isopropanol [22].

## Discussion

### Overview

This scoping review shows that smart glasses have both benefits and limitations in complex care environments. Increased understanding is provided about different situations where smart glasses might be used by health care professionals in clinical practice in anesthesia departments and ICUs. Research about smart glasses in clinical complex care environments is limited; several of the included studies were conducted in simulated settings or were minor clinical studies. The results also show that smart glasses could affect health care professionals and their performance, both positively (eg, through increased focus on procedural fields) and negatively (eg, causing discomfort during use). The quality of use of smart glasses is highlighted and there are some concerns that need attention before implementing the use of smart glasses in clinical complex care. This is all useful knowledge in the process of implementing smart glasses in anesthesia departments and ICUs.

### Principal Results and Comparison With Prior Work

In complex care environments, technology is a prerequisite for the advanced care conducted. When health care professionals feel confident with equipment, complex care can be carried out in a safe way [47]. This review shows that patient management deteriorated if health care professionals became disturbed by the smart glasses. The results further highlight that there was a learning curve associated with the use of smart glasses. This indicates that user training is crucial when introducing smart glasses into complex care environments in order to maintain high-quality care and patient safety. The same is true for prudent implementation of any new technology. Both of these aspects have been discussed in relation to ICUs [21] and anesthesia departments seem to adhere to this as well. Generally, when implementing new technology, health care professionals need to see a clear benefit with the new device [48], and implementation in complex care environments does not seem to be an exception. To ensure patient privacy and patient safety, this review shows that ethical issues also need to be taken into consideration before implementing the use of smart glasses into complex care environments, as well as in other contexts [13,15,17,49]. Information security and privacy are well-known issues when implementing eHealth solutions in health care [50], and cybersecurity is at the top of ECRI’s annual list of patient safety risks for 2019 [6]. Research regarding cybersecurity in health care has increased over the last 20 years, but there are still gaps to fill [51]. An extensive review about ethical issues related to smart glasses states that data security and privacy were the most frequently highlighted features found in the research [52]. Smart glasses as a new platform also imposes new ethical challenges related to privacy (eg, it is impossible for patients and health care professionals to know if or when they are being recorded or photographed by smart glasses). This makes context-specific development, implementation, and user routines important from an ethical view in order to provide patient safety [52]. Both intended and unintended consequences of new technology, such as smart glasses, need to be taken into account in the process of implementation [10], for example, in complex care environments.

The results show that smart glasses are versatile tools that could be used for several situations occurring in complex care environments. Patient vital signs are one important part of surveillance in complex care environments that are used to detect early changes in patients’ conditions that might need urgent and immediate attention. This review shows that smart glasses presenting vital signs made health care professionals detect abnormal vital signs faster. The results also reveal that health care professionals did not have to turn their heads away from the patients in order to view monitors. This has been shown earlier in complex care environments with other types of more cumbersome head-mounted displays [53-55] and with smart glasses in surgical settings [17]. Not having to turn one’s head away from the procedural field has been suggested to increase patient safety [53]. Further, the results show that smart glasses provided the possibility for uninterrupted monitoring when health care professionals needed to leave the traditional monitor out of sight. This is in line with earlier research conducted in surgical settings [49] and has been seen as a valuable asset for increased patient safety [21].

This review indicates that infrastructure, smart glasses’ performance, and health care professionals will affect the usability of smart glasses in complex care environments. Infrastructure, such as Wi-Fi and streaming, is a prerequisite for clinical use of most new technology [50], including smart glasses, and has been found to be a limitation in both surgical and nonsurgical settings [15,18]. This review found that complex care environments are no exception. Other technical limitations, including battery life and heat generation, were found in this review and are well known [13,15,17,49]. Technical improvements have been made recently [56], but no research was found using new, improved smart glasses. This review shows that the quality of photos and video captured by smart glasses seems to be sufficient for most clinical uses in complex care environments, but not for interpreting medical images with subtle findings. This has been concluded in the past for surgical settings [19], although other reviews have found photo and video quality to be a clinical limitation in various settings [13,15]. In complex care environments, monitoring vital signs in real time is one area of use for smart glasses, and this review found no negative results regarding image quality or the ability to detect abnormalities when smart glasses were used for viewing this kind of information.

This review shows that research about smart glasses in clinical complex care environments is limited. The results from this review can provide valuable knowledge to meet the growing interest from health care professionals, product developers, and researchers concerning smart glasses and their possible implementation in complex care environments.

### Methodological Considerations and Limitations

Since a scoping review aims to conduct a wide rather than in-depth synthesis of research [25], PubMed and Scopus were chosen to search for articles from both health care and engineering. Perhaps more articles would have been found if more databases had been used, but PubMed and Scopus are big databases with wide coverage and were found sufficient. The search and screening processes were performed by the first author (CR) with support from experienced librarians. It is possible that relevant articles were missed and that these would have been found if this process had been performed by more than one researcher [27]. After reading the obtained full-text articles, inclusions and exclusions were discussed among the authors until consensus was reached. In the initial searches, “Google Glass” was found as a keyword, hence we chose to add this phrase as a search term. If we had added other brand names, we might have found additional articles, but Google Glass is the most well-known brand of smart glasses. Articles focusing on surgeons’ use of smart glasses were not actively searched for; however, those found using our search terms were included if the inclusion criteria were met. The surgeons’ focus was assumed to be mainly on the surgical field, but they often work closely with an anesthesiologist or a nurse anesthetist. It is possible that issues such as team communication or other applicable information were addressed in articles with a surgical focus, which could have added to our study as well. Reviews can be accepted in a scoping review, but we chose to exclude them since there is a risk of bias when interpreting other researchers’ interpretations. *Gray literature*, such as dissertations and books, can also be included in a scoping review [25]. We found some gray literature during our searches (eg, correspondence and nonscientific articles) and they were included if inclusion criteria were met. Furthermore, a scoping review does not seek to assess the quality or impact of the results from the articles included [24,25]. This is why no quality assessment was made during the inclusion process. Articles with both qualitative and quantitative designs were included in our study, and text from results and tables were included in the analysis. The analysis process was discussed among the authors to increase credibility. The sixth optional stage in the scoping process (ie, step 6), where practitioners and consumers were included [25], added value to the study through creative discussions and input. After conducting this study, the authors conclude that a scoping review was suitable to fulfill the research objective.

### Conclusions

Smart glasses were found to be both a helpful tool and a hindrance in caring situations that might occur in complex care environments. Thoughtful implementation and improved hardware are needed to meet health care professionals’ needs. It has been stated earlier that all new technology brings new errors, and that new technologies should be tested before widespread implementation [7]. New technology might also bring ethical challenges [52]. Therefore, we conclude that more research is required to elucidate how smart glasses affect patient safety, health care professionals, and quality of care in complex care environments.

## Appendix

Multimedia Appendix 1 Final search terms.

Multimedia Appendix 2 Presentation of database searches.

Multimedia Appendix 3 Charting of articles included in the qualitative content analysis.

## Abbreviations

CPR: cardiopulmonary resuscitation
ECG: electrocardiogram
ECRI: Emergency Care Research Institute
ICU: intensive care unit
PRISMA: Preferred Reporting Items for Systematic Reviews and Meta-Analyses
PRISMA-ScR: Preferred Reporting Items for Systematic Reviews and Meta-Analyses extension for scoping reviews
