# Supplementary material for: Smart Glasses for Caring Situations in Complex Care Environments: Scoping Review
Source: JMIR Mhealth Uhealth. 2020 Apr 20;8(4):e16055. doi: 10.2196/16055 (PMC7199139; doi:10.2196/16055)
Supplement: Multimedia Appendix 1 [file mhealth_v8i4e16055_app1.docx]

## Appendix 1: Final search terms

* Is used for truncation in PubMed and SCOPUS.

| Main area | PubMed | SCOPUS |
| --- | --- | --- |
| Smart Glasses | Eyeglasses [Mesh], Eyeglasses, Eyeglas*, Glass, Glasses, Smart Glass, Smart Glasses, Google glass, Google glasses, Head up display, Prism glass, Prism glasses | Eyeglasses, Eyeglass, Eyeglas*, Glass, Glasses, Smart Glass, Smart Glasses, Google glass, Google glasses, Head up display, Prism glass* |
| Intensive care | Critical care [Mesh], Critical care, Intensive care units [Mesh], Intensive care units, Critical care nursing [Mesh], Critical care nursing, ICU, Intensiv*, Critical* | Critical care, Intensive care units, Intensive care unit, Critical care nursing, ICU, Intensive care, Intensiv*, Critical* |
| Anesthesiology | Anesthesiologists [Mesh], Anesthesiologists, Anesthesiology [Mesh], Anesthesiology, Anesthesia department hospital [Mesh], Anesthesia department hospital, Nurse anesthetists [Mesh], Nurse anesthetists, Anesthetists [Mesh], Anesthetists, Anesthe*, Anaesthe* | Anesthesiologists, Anesthesiology , Anesthesia department hospital, Nurse anesthetists, Anesthetists, Anesthe*, Anaesthe* |
| Alarm management related to vital signs | Clinical alarms [Mesh], Clinical alarm, Monitoring physiologic [Mesh], Monitoring physiologic, Vital signs [Mesh], Vital signs, Medical order entry systems [Mesh], Medical order entry systems, Monitor*, Alarm*, Vital*, Alert*, Hands free, Head mounted display | Clinical alarm, Monitoring physiologic, Vital signs, Medical order entry systems, Monitor*, Alarm*, Vital*, Alert*, Hands free, Head mounted display |
| Patient safety | Patient safety [Mesh], Patient safety | Patient safety |
