# Supplementary material for: Smart Glasses for Caring Situations in Complex Care Environments: Scoping Review
Source: JMIR Mhealth Uhealth. 2020 Apr 20;8(4):e16055. doi: 10.2196/16055 (PMC7199139; doi:10.2196/16055)
Supplement: Multimedia Appendix 2 [file mhealth_v8i4e16055_app2.docx]

## Appendix 2: Presentation of database searches

### Searches in PubMed.

Searches were made in “all fields”. If too many items were found, limits were set to title/abstract to see if this generated a more reasonable amount of hits. No limits were set to “article type”.

* Is used for truncation in PubMed.

| Query | Items  found | Full-  text |
| --- | --- | --- |
| **Searches in context “smart glasses”, 2018-03-05 to 2018-03-08** | | |
| Search "Eyeglasses"[Mesh] Sort by: Best Match | 7204 | - |
| Search Eyeglasses Sort by: Best Match | 7568 | - |
| Search glass Sort by: Best Match | 105778 | - |
| Search glasses Sort by: Best Match | 95834 | - |
| Search "smart glass" Sort by: Best Match | 19 | 1 |
| Search "smart glasses" Sort by: Best Match | 32 | 4 |
| Search "google glass" Sort by: Best Match | 136 | 26 |
| Search "google glasses" Sort by: Best Match | 2 | 0 |
| Search (("Eyeglasses"[Mesh]) OR Eyeglasses) OR eyeglas | 7555 | - |
| Search eyeglas* | 7555 | - |
| Search "prism glasses" | 80 | 0 |
| Search "prism glass" | 5 | 0 |
| Search "head up display" | 65 | 2 |
| **Searches in context ”intensive care” 2018-03-05 to 2018-03-08** | | |
| Search "Critical Care"[Mesh] | 51378 | - |
| Search "Intensive Care Units"[Mesh] | 71123 | - |
| Search "Critical Care Nursing"[Mesh] | 1262 | - |
| Search Critical Care | 211087 | - |
| Search Intensive Care Units | 85673 | - |
| Search Critical Care Nursing | 26867 | - |
| Search ICU | 110148 | - |
| Search critical* | 792862 | - |
| Search intensiv* | 331718 | - |
| Search (((((((("Critical Care"[Mesh]) OR "Intensive Care Units"[Mesh]) OR "Critical Care Nursing"[Mesh]) OR Critical Care) OR Intensive Care Units) OR Critical Care Nursing) OR ICU) OR critical*) OR intensiv* | 1069395 | - |
| Search (((((((((("Critical Care"[Mesh]) OR "Intensive Care Units"[Mesh]) OR "Critical Care Nursing"[Mesh]) OR Critical Care) OR Intensive Care Units) OR Critical Care Nursing) OR ICU) OR critical*) OR intensiv*)) AND ((("Eyeglasses"[Mesh]) OR Eyeglasses) OR eyeglass*) | 108 | 2 |
| Search (((((((((("Critical Care"[Mesh]) OR "Intensive Care Units"[Mesh]) OR "Critical Care Nursing"[Mesh]) OR Critical Care) OR Intensive Care Units) OR Critical Care Nursing) OR ICU) OR critical*) OR intensiv*)) AND glass | 4122 | - |
| Search (((((((((("Critical Care"[Mesh]) OR "Intensive Care Units"[Mesh]) OR "Critical Care Nursing"[Mesh]) OR Critical Care) OR Intensive Care Units) OR Critical Care Nursing) OR ICU) OR critical*) OR intensiv*)) AND glass[Title/Abstract] | 3503 | - |
| Search (((((((((("Critical Care"[Mesh]) OR "Intensive Care Units"[Mesh]) OR "Critical Care Nursing"[Mesh]) OR Critical Care) OR Intensive Care Units) OR Critical Care Nursing) OR ICU) OR critical*) OR intensiv*)) AND glasses | 577 | - |
| Search (((((((((("Critical Care"[Mesh]) OR "Intensive Care Units"[Mesh]) OR "Critical Care Nursing"[Mesh]) OR Critical Care) OR Intensive Care Units) OR Critical Care Nursing) OR ICU) OR critical*) OR intensiv*)) AND glasses[Title/Abstract] | 491 | - |
| Search (((((((((("Critical Care"[Mesh]) OR "Intensive Care Units"[Mesh]) OR "Critical Care Nursing"[Mesh]) OR Critical Care) OR Intensive Care Units) OR Critical Care Nursing) OR ICU) OR critical*) OR intensiv*)) AND "smart glass" | 2 | 1 |
| Search (((((((((("Critical Care"[Mesh]) OR "Intensive Care Units"[Mesh]) OR "Critical Care Nursing"[Mesh]) OR Critical Care) OR Intensive Care Units) OR Critical Care Nursing) OR ICU) OR critical*) OR intensiv*)) AND "smart glasses" | 4 | 2 |
| Search (((((((((("Critical Care"[Mesh]) OR "Intensive Care Units"[Mesh]) OR "Critical Care Nursing"[Mesh]) OR Critical Care) OR Intensive Care Units) OR Critical Care Nursing) OR ICU) OR critical*) OR intensiv*)) AND "google glass" | 15 | 6 |
| Search (((((((((("Critical Care"[Mesh]) OR "Intensive Care Units"[Mesh]) OR "Critical Care Nursing"[Mesh]) OR Critical Care) OR Intensive Care Units) OR Critical Care Nursing) OR ICU) OR critical*) OR intensiv*)) AND "google glasses" | 0 | - |
| Search (((((((((("Critical Care"[Mesh]) OR "Intensive Care Units"[Mesh]) OR "Critical Care Nursing"[Mesh]) OR Critical Care) OR Intensive Care Units) OR Critical Care Nursing) OR ICU) OR critical*) OR intensiv*)) AND "head up display" | 10 | 0 |
| **Searches in context “anaesthesiology” 2018-03-05 to 2018-03-08** | | |
| Search "Anesthesiologists"[Mesh] | 281 | - |
| Search "Anesthesiology"[Mesh] | 29578 | - |
| Search "Anesthesia Department, Hospital"[Mesh] | 1031 | - |
| Search "Nurse Anesthetists"[Mesh] | 2526 | - |
| Search "Anesthetists"[Mesh] | 2834 | - |
| Search anesthesia departments, hospital | 54251 | - |
| Search Anesthesiologists | 13260 | - |
| Search Anesthesiology | 180205 | - |
| Search Nurse Anesthetists | 2799 | - |
| Search Anesthetists | 4234 | - |
| Search anesthe* | 583303 | - |
| Search anaesthe* | 168764 | - |
| Search ((((((((((("Anesthesiologists"[Mesh]) OR "Anesthesiology"[Mesh]) OR "Anesthesia Department, Hospital"[Mesh]) OR "Nurse Anesthetists"[Mesh]) OR "Anesthetists"[Mesh]) OR Anesthesiologists) OR Anesthesiology) OR anesthesia departments, hospital) OR Nurse Anesthetists) OR Anesthetists) OR anesthe*) OR anaesthe* | 671599 | - |
| Search ((((((((((((("Anesthesiologists"[Mesh]) OR "Anesthesiology"[Mesh]) OR "Anesthesia Department, Hospital"[Mesh]) OR "Nurse Anesthetists"[Mesh]) OR "Anesthetists"[Mesh]) OR Anesthesiologists) OR Anesthesiology) OR anesthesia departments, hospital) OR Nurse Anesthetists) OR Anesthetists) OR anesthe*) OR anaesthe*)) AND ((("Eyeglasses"[Mesh]) OR Eyeglasses) OR eyeglas*) | 41 | - |
| Search ((((((((((((("Anesthesiologists"[Mesh]) OR "Anesthesiology"[Mesh]) OR "Anesthesia Department, Hospital"[Mesh]) OR "Nurse Anesthetists"[Mesh]) OR "Anesthetists"[Mesh]) OR Anesthesiologists) OR Anesthesiology) OR anesthesia departments, hospital) OR Nurse Anesthetists) OR Anesthetists) OR anesthe*) OR anaesthe*)) AND glass | 1829 | - |
| Search ((((((((((((("Anesthesiologists"[Mesh]) OR "Anesthesiology"[Mesh]) OR "Anesthesia Department, Hospital"[Mesh]) OR "Nurse Anesthetists"[Mesh]) OR "Anesthetists"[Mesh]) OR Anesthesiologists) OR Anesthesiology) OR anesthesia departments, hospital) OR Nurse Anesthetists) OR Anesthetists) OR anesthe*) OR anaesthe*)) AND glass[Title/Abstract] | 1463 | - |
| Search ((((((((((((("Anesthesiologists"[Mesh]) OR "Anesthesiology"[Mesh]) OR "Anesthesia Department, Hospital"[Mesh]) OR "Nurse Anesthetists"[Mesh]) OR "Anesthetists"[Mesh]) OR Anesthesiologists) OR Anesthesiology) OR anesthesia departments, hospital) OR Nurse Anesthetists) OR Anesthetists) OR anesthe*) OR anaesthe*)) AND glasses | 111 | 1 |
| Search ((((((((((((("Anesthesiologists"[Mesh]) OR "Anesthesiology"[Mesh]) OR "Anesthesia Department, Hospital"[Mesh]) OR "Nurse Anesthetists"[Mesh]) OR "Anesthetists"[Mesh]) OR Anesthesiologists) OR Anesthesiology) OR anesthesia departments, hospital) OR Nurse Anesthetists) OR Anesthetists) OR anesthe*) OR anaesthe*)) AND "smart glass" | 0 | - |
| Search ((((((((((((("Anesthesiologists"[Mesh]) OR "Anesthesiology"[Mesh]) OR "Anesthesia Department, Hospital"[Mesh]) OR "Nurse Anesthetists"[Mesh]) OR "Anesthetists"[Mesh]) OR Anesthesiologists) OR Anesthesiology) OR anesthesia departments, hospital) OR Nurse Anesthetists) OR Anesthetists) OR anesthe*) OR anaesthe*)) AND "smart glasses" | 0 | - |
| Search ((((((((((((("Anesthesiologists"[Mesh]) OR "Anesthesiology"[Mesh]) OR "Anesthesia Department, Hospital"[Mesh]) OR "Nurse Anesthetists"[Mesh]) OR "Anesthetists"[Mesh]) OR Anesthesiologists) OR Anesthesiology) OR anesthesia departments, hospital) OR Nurse Anesthetists) OR Anesthetists) OR anesthe*) OR anaesthe*)) AND "google glass" | 3 | 3 |
| Search ((((((((((((("Anesthesiologists"[Mesh]) OR "Anesthesiology"[Mesh]) OR "Anesthesia Department, Hospital"[Mesh]) OR "Nurse Anesthetists"[Mesh]) OR "Anesthetists"[Mesh]) OR Anesthesiologists) OR Anesthesiology) OR anesthesia departments, hospital) OR Nurse Anesthetists) OR Anesthetists) OR anesthe*) OR anaesthe*)) AND "google glasses" | 0 | - |
| Search ((((((((((((("Anesthesiologists"[Mesh]) OR "Anesthesiology"[Mesh]) OR "Anesthesia Department, Hospital"[Mesh]) OR "Nurse Anesthetists"[Mesh]) OR "Anesthetists"[Mesh]) OR Anesthesiologists) OR Anesthesiology) OR anesthesia departments, hospital) OR Nurse Anesthetists) OR Anesthetists) OR anesthe*) OR anaesthe*)) AND "head up display" | 2 | 2 |
| **Searches in context “alarm management” and “vital signs” 2018-04-16** | | |
| Search "Clinical Alarms"[Mesh] | 633 | - |
| Search "Clinical Alarms" | 664 | - |
| Search "Monitoring, Physiologic"[Mesh] | 156747 | - |
| Search "Monitoring, Physiologic" | 50969 | - |
| Search "Vital Signs"[Mesh] | 386845 | - |
| Search "Vital Signs" | 10723 | - |
| Search "Medical Order Entry Systems"[Mesh] | 1997 | - |
| Search "Medical Order Entry Systems" | 2035 | - |
| Search monitor* | 836337 | - |
| Search alarm* | 23546 | - |
| Search vital* | 155628 | - |
| Search alert* | 37975 | - |
| Search "hands free" | 524 | - |
| Search "head mounted display" | 461 | - |
| Search ((((((((((("Clinical Alarms"[Mesh]) OR "Clinical Alarms") OR "Monitoring, Physiologic"[Mesh]) OR "Monitoring, Physiologic") OR "Vital Signs"[Mesh]) OR "Vital Signs") OR "Medical Order Entry Systems"[Mesh]) OR "Medical Order Entry Systems") OR monitor*) OR alarm*) OR vital*) OR alert* | 1405791 | - |
| Search ((((((((((((("Clinical Alarms"[Mesh]) OR "Clinical Alarms") OR "Monitoring, Physiologic"[Mesh]) OR "Monitoring, Physiologic") OR "Vital Signs"[Mesh]) OR "Vital Signs") OR "Medical Order Entry Systems"[Mesh]) OR "Medical Order Entry Systems") OR monitor*) OR alarm*) OR vital*) OR alert*)) AND ((("Eyeglasses"[Mesh]) OR "Eyeglasses") OR Eyeglas*) | 214 | 5 |
| Search ((((((((((((("Clinical Alarms"[Mesh]) OR "Clinical Alarms") OR "Monitoring, Physiologic"[Mesh]) OR "Monitoring, Physiologic") OR "Vital Signs"[Mesh]) OR "Vital Signs") OR "Medical Order Entry Systems"[Mesh]) OR "Medical Order Entry Systems") OR monitor*) OR alarm*) OR vital*) OR alert*)) AND glass | 6041 | - |
| Search ((((((((((((("Clinical Alarms"[Mesh]) OR "Clinical Alarms") OR "Monitoring, Physiologic"[Mesh]) OR "Monitoring, Physiologic") OR "Vital Signs"[Mesh]) OR "Vital Signs") OR "Medical Order Entry Systems"[Mesh]) OR "Medical Order Entry Systems") OR monitor*) OR alarm*) OR vital*) OR alert*)) AND glass[Title/Abstract] | 5222 | - |
| Search ((((((((((((("Clinical Alarms"[Mesh]) OR "Clinical Alarms") OR "Monitoring, Physiologic"[Mesh]) OR "Monitoring, Physiologic") OR "Vital Signs"[Mesh]) OR "Vital Signs") OR "Medical Order Entry Systems"[Mesh]) OR "Medical Order Entry Systems") OR monitor*) OR alarm*) OR vital*) OR alert*)) AND glasses | 583 | - |
| Search ((((((((((((("Clinical Alarms"[Mesh]) OR "Clinical Alarms") OR "Monitoring, Physiologic"[Mesh]) OR "Monitoring, Physiologic") OR "Vital Signs"[Mesh]) OR "Vital Signs") OR "Medical Order Entry Systems"[Mesh]) OR "Medical Order Entry Systems") OR monitor*) OR alarm*) OR vital*) OR alert*)) AND glasses[Title/Abstract] | 434 | - |
| Search ((((((((((((("Clinical Alarms"[Mesh]) OR "Clinical Alarms") OR "Monitoring, Physiologic"[Mesh]) OR "Monitoring, Physiologic") OR "Vital Signs"[Mesh]) OR "Vital Signs") OR "Medical Order Entry Systems"[Mesh]) OR "Medical Order Entry Systems") OR monitor*) OR alarm*) OR vital*) OR alert*)) AND "smart glass" | 4 | 1 |
| Search ((((((((((((("Clinical Alarms"[Mesh]) OR "Clinical Alarms") OR "Monitoring, Physiologic"[Mesh]) OR "Monitoring, Physiologic") OR "Vital Signs"[Mesh]) OR "Vital Signs") OR "Medical Order Entry Systems"[Mesh]) OR "Medical Order Entry Systems") OR monitor*) OR alarm*) OR vital*) OR alert*)) AND "smart glasses" | 9 | 1 |
| Search ((((((((((((("Clinical Alarms"[Mesh]) OR "Clinical Alarms") OR "Monitoring, Physiologic"[Mesh]) OR "Monitoring, Physiologic") OR "Vital Signs"[Mesh]) OR "Vital Signs") OR "Medical Order Entry Systems"[Mesh]) OR "Medical Order Entry Systems") OR monitor*) OR alarm*) OR vital*) OR alert*)) AND "google glass" | 21 | 7 |
| Search ((((((((((((("Clinical Alarms"[Mesh]) OR "Clinical Alarms") OR "Monitoring, Physiologic"[Mesh]) OR "Monitoring, Physiologic") OR "Vital Signs"[Mesh]) OR "Vital Signs") OR "Medical Order Entry Systems"[Mesh]) OR "Medical Order Entry Systems") OR monitor*) OR alarm*) OR vital*) OR alert*)) AND "google glasses" | 0 | - |
| Search ((((((((((((("Clinical Alarms"[Mesh]) OR "Clinical Alarms") OR "Monitoring, Physiologic"[Mesh]) OR "Monitoring, Physiologic") OR "Vital Signs"[Mesh]) OR "Vital Signs") OR "Medical Order Entry Systems"[Mesh]) OR "Medical Order Entry Systems") OR monitor*) OR alarm*) OR vital*) OR alert*)) AND "head up display" | 15 | 2 |
| Search ((((((((((((("Clinical Alarms"[Mesh]) OR "Clinical Alarms") OR "Monitoring, Physiologic"[Mesh]) OR "Monitoring, Physiologic") OR "Vital Signs"[Mesh]) OR "Vital Signs") OR "Medical Order Entry Systems"[Mesh]) OR "Medical Order Entry Systems") OR monitor*) OR alarm*) OR vital*) OR alert*)) AND "hands free" | 68 | 3 |
| Search ((((((((((((("Clinical Alarms"[Mesh]) OR "Clinical Alarms") OR "Monitoring, Physiologic"[Mesh]) OR "Monitoring, Physiologic") OR "Vital Signs"[Mesh]) OR "Vital Signs") OR "Medical Order Entry Systems"[Mesh]) OR "Medical Order Entry Systems") OR monitor*) OR alarm*) OR vital*) OR alert*)) AND "head mounted display" | 78 | 4 |
| **Searches in context ”patient safety” 2018-04-05** | | |
| Search "Patient Safety"[Mesh] | 13971 | - |
| Search "Patient Safety" | 35750 | - |
| Search (("Eyeglasses"[Mesh]) OR Eyeglasses) OR Eyeglas* | 7567 | - |
| Search ("Patient Safety"[Mesh]) OR "Patient Safety" | 35750 | - |
| Search ((("Patient Safety"[Mesh]) OR "Patient Safety")) AND ((("Eyeglasses"[Mesh]) OR Eyeglasses) OR Eyeglas*) | 5 | 1 |
| Search ((("Patient Safety"[Mesh]) OR "Patient Safety")) AND glass | 48 | 1 |
| Search ((("Patient Safety"[Mesh]) OR "Patient Safety")) AND glasses | 18 | 2 |
| Search ((("Patient Safety"[Mesh]) OR "Patient Safety")) AND "smart glass" | 0 | - |
| Search ((("Patient Safety"[Mesh]) OR "Patient Safety")) AND "smart glasses" | 1 | 1 |
| Search ((("Patient Safety"[Mesh]) OR "Patient Safety")) AND "google glass" | 5 | 1 |
| Search ((("Patient Safety"[Mesh]) OR "Patient Safety")) AND "google glasses" | 0 | - |
| Search ((("Patient Safety"[Mesh]) OR "Patient Safety")) AND "head up display" | 1 | 0 |

### Searches in SCOPUS.

Searches were made in title, abstract and keywords. No limits were set.

* Is used for truncation in SCOPUS.

| Query | Items found | Full-text |
| --- | --- | --- |
| **Searches in context ”smart glasses”, 2018-04-10 and 04-16** | | |
| TITLE-ABS-KEY ( eyeglasses ) | 8,202 | - |
| TITLE-ABS-KEY ( eyeglass ) | 8,202 | - |
| TITLE-ABS-KEY ( eyeglas* ) | 8,205 | - |
| TITLE-ABS-KEY ( glass ) | 607,007 | - |
| TITLE-ABS-KEY ( glasses ) | 607,007 | - |
| TITLE-ABS-KEY ( "smart glass" ) | 524 | - |
| TITLE-ABS-KEY ( "smart glasses" ) | 524 | - |
| TITLE-ABS-KEY ( "google glass" ) | 611 | - |
| TITLE-ABS-KEY ( "google glasses" ) | 611 | - |
| TITLE-ABS-KEY ( "head up display" ) | 1,724 | - |
| TITLE-ABS-KEY ( "prism glass*" )  Limits = Excluding all articles about Ophthalmology, aviation, radiology and dentistry.  Since Prism glass* did not generate any relevant items, this search term was not used in the following searches. | 174  After limits:  116 | 0 |
| **Searches in context ”intensive care”, 2018-04-10** | | |
| TITLE-ABS-KEY ( "intensive care" ) | 253,134 | - |
| TITLE-ABS-KEY ( icu ) | 54,511 | - |
| TITLE-ABS-KEY ( "critical care" ) | 60,610 | - |
| TITLE-ABS-KEY ( "critical care nursing" ) | 2,437 | - |
| TITLE-ABS-KEY ( "intensive care units" ) | 172,991 | - |
| TITLE-ABS-KEY ( "intensive care unit" ) | 172,991 | - |
| TITLE-ABS-KEY ( critical* ) | 1,971,465 | - |
| TITLE-ABS-KEY ( intensiv* ) | 575,860 | - |
| ( ( TITLE-ABS-KEY ( "intensive care" ) )  OR  ( TITLE-ABS-KEY ( icu ) )  OR  ( TITLE-ABS-KEY ( "critical care" ) )  OR  ( TITLE-ABS-KEY ( "critical care nursing" ) )  OR  ( TITLE-ABS-KEY ( "intensive care unit" ) )  OR  ( TITLE-ABS-KEY ( critical* ) )  OR  ( TITLE-ABS-KEY ( intensiv* ) ) )  AND  ( ( TITLE-ABS-KEY ( "patient safety" ) )  AND  ( ( TITLE-ABS-KEY ( eyeglasses ) )  OR  ( TITLE-ABS-KEY ( eyeglass ) )  OR  ( TITLE-ABS-KEY ( eyeglas* ) ) ) ) | 2 | 1 |
| ( ( TITLE-ABS-KEY ( "intensive care" ) )  OR  ( TITLE-ABS-KEY ( icu ) )  OR  ( TITLE-ABS-KEY ( "critical care" ) )  OR  ( TITLE-ABS-KEY ( "critical care nursing" ) )  OR  ( TITLE-ABS-KEY ( "intensive care unit" ) )  OR  ( TITLE-ABS-KEY ( critical* ) )  OR  ( TITLE-ABS-KEY ( intensiv* ) ) )  AND  ( TITLE-ABS-KEY ( glass ) ) | 24,185 | - |
| ( ( TITLE-ABS-KEY ( "intensive care" ) )  OR  ( TITLE-ABS-KEY ( icu ) )  OR  ( TITLE-ABS-KEY ( "critical care" ) )  OR  ( TITLE-ABS-KEY ( "critical care nursing" ) )  OR  ( TITLE-ABS-KEY ( "intensive care unit" ) )  OR  ( TITLE-ABS-KEY ( critical* ) )  OR  ( TITLE-ABS-KEY ( intensiv* ) ) )  AND  ( TITLE-ABS-KEY ( glasses ) ) | 24,185 | - |
| ( ( TITLE-ABS-KEY ( "intensive care" ) )  OR  ( TITLE-ABS-KEY ( icu ) )  OR  ( TITLE-ABS-KEY ( "critical care" ) )  OR  ( TITLE-ABS-KEY ( "critical care nursing" ) )  OR  ( TITLE-ABS-KEY ( "intensive care unit" ) )  OR  ( TITLE-ABS-KEY ( critical* ) )  OR  ( TITLE-ABS-KEY ( intensiv* ) ) )  AND  ( TITLE-ABS-KEY ( "smart glass" ) ) | 27 | 2 |
| ( ( TITLE-ABS-KEY ( "intensive care" ) )  OR  ( TITLE-ABS-KEY ( icu ) )  OR  ( TITLE-ABS-KEY ( "critical care" ) )  OR  ( TITLE-ABS-KEY ( "critical care nursing" ) )  OR  ( TITLE-ABS-KEY ( "intensive care unit" ) )  OR  ( TITLE-ABS-KEY ( critical* ) )  OR  ( TITLE-ABS-KEY ( intensiv* ) ) )  AND  ( TITLE-ABS-KEY ( "smart glasses" ) ) | 27 | 2 |
| ( ( TITLE-ABS-KEY ( "intensive care" ) )  OR  ( TITLE-ABS-KEY ( icu ) )  OR  ( TITLE-ABS-KEY ( "critical care" ) )  OR  ( TITLE-ABS-KEY ( "critical care nursing" ) )  OR  ( TITLE-ABS-KEY ( "intensive care unit" ) )  OR  ( TITLE-ABS-KEY ( critical* ) )  OR  ( TITLE-ABS-KEY ( intensiv* ) ) )  AND  ( TITLE-ABS-KEY ( "google glass" ) ) | 30 | 6 |
| ( ( TITLE-ABS-KEY ( "intensive care" ) )  OR  ( TITLE-ABS-KEY ( icu ) )  OR  ( TITLE-ABS-KEY ( "critical care" ) )  OR  ( TITLE-ABS-KEY ( "critical care nursing" ) )  OR  ( TITLE-ABS-KEY ( "intensive care unit" ) )  OR  ( TITLE-ABS-KEY ( critical* ) )  OR  ( TITLE-ABS-KEY ( intensiv* ) ) )  AND  ( TITLE-ABS-KEY ( "google glasses" ) ) | 30 | 6 |
| ( ( TITLE-ABS-KEY ( "intensive care" ) )  OR  ( TITLE-ABS-KEY ( icu ) )  OR  ( TITLE-ABS-KEY ( "critical care" ) )  OR  ( TITLE-ABS-KEY ( "critical care nursing" ) )  OR  ( TITLE-ABS-KEY ( "intensive care unit" ) )  OR  ( TITLE-ABS-KEY ( critical* ) )  OR  ( TITLE-ABS-KEY ( intensiv* ) ) )  AND  ( TITLE-ABS-KEY ( "head up display" ) ) | 116 | 0 |
| **Searches in context ”anastesiology”, 2018-04-12** | | |
| TITLE-ABS-KEY ( anesthesiologists ) | 31,473 | - |
| TITLE-ABS-KEY ( anesthesiology ) | 30,960 | - |
| TITLE-ABS-KEY ( "anesthesia department hospital" ) | 963 | - |
| TITLE-ABS-KEY ( "nurse anesthetists" ) | 3,291 | - |
| TITLE-ABS-KEY ( anesthetists ) | 6,673 | - |
| TITLE-ABS-KEY ( anesthe* ) | 546,237 | - |
| TITLE-ABS-KEY ( anaesthe* ) | 142,117 | - |
| ( ( TITLE-ABS-KEY ( anesthesiologists ) )  OR  ( TITLE-ABS-KEY ( anesthesiology ) )  OR  ( TITLE-ABS-KEY ( "anesthesia department hospital" ) )  OR  ( TITLE-ABS-KEY ( "nurse anesthetists" ) )  OR  ( TITLE-ABS-KEY ( anesthetists ) )  OR  ( TITLE-ABS-KEY ( anesthe* ) )  OR  ( TITLE-ABS-KEY ( anaesthe* ) ) )  AND  ( ( TITLE-ABS-KEY ( eyeglasses ) )  OR  ( TITLE-ABS-KEY ( eyeglass ) )  OR  ( TITLE-ABS-KEY ( eyeglas* ) ) ) | 43 | 0 |
| ( ( TITLE-ABS-KEY ( anesthesiologists ) )  OR  ( TITLE-ABS-KEY ( anesthesiology ) )  OR  ( TITLE-ABS-KEY ( "anesthesia department hospital" ) )  OR  ( TITLE-ABS-KEY ( "nurse anesthetists" ) )  OR  ( TITLE-ABS-KEY ( anesthetists ) )  OR  ( TITLE-ABS-KEY ( anesthe* ) )  OR  ( TITLE-ABS-KEY ( anaesthe* ) ) )  AND  ( TITLE-ABS-KEY ( glass ) ) | 1,622 | - |
| ( ( TITLE-ABS-KEY ( anesthesiologists ) )  OR  ( TITLE-ABS-KEY ( anesthesiology ) )  OR  ( TITLE-ABS-KEY ( "anesthesia department hospital" ) )  OR  ( TITLE-ABS-KEY ( "nurse anesthetists" ) )  OR  ( TITLE-ABS-KEY ( anesthetists ) )  OR  ( TITLE-ABS-KEY ( anesthe* ) )  OR  ( TITLE-ABS-KEY ( anaesthe* ) ) )  AND  ( TITLE-ABS-KEY ( glasses ) ) | 1,622 | - |
| ( ( TITLE-ABS-KEY ( anesthesiologists ) )  OR  ( TITLE-ABS-KEY ( anesthesiology ) )  OR  ( TITLE-ABS-KEY ( "anesthesia department hospital" ) )  OR  ( TITLE-ABS-KEY ( "nurse anesthetists" ) )  OR  ( TITLE-ABS-KEY ( anesthetists ) )  OR  ( TITLE-ABS-KEY ( anesthe* ) )  OR  ( TITLE-ABS-KEY ( anaesthe* ) ) )  AND  ( TITLE-ABS-KEY ( "smart glass" ) ) | 0 | 0 |
| ( ( TITLE-ABS-KEY ( anesthesiologists ) )  OR  ( TITLE-ABS-KEY ( anesthesiology ) )  OR  ( TITLE-ABS-KEY ( "anesthesia department hospital" ) )  OR  ( TITLE-ABS-KEY ( "nurse anesthetists" ) )  OR  ( TITLE-ABS-KEY ( anesthetists ) )  OR  ( TITLE-ABS-KEY ( anesthe* ) )  OR  ( TITLE-ABS-KEY ( anaesthe* ) ) )  AND  ( TITLE-ABS-KEY ( "smart glasses" ) ) | 0 | 0 |
| ( ( TITLE-ABS-KEY ( anesthesiologists ) )  OR  ( TITLE-ABS-KEY ( anesthesiology ) )  OR  ( TITLE-ABS-KEY ( "anesthesia department hospital" ) )  OR  ( TITLE-ABS-KEY ( "nurse anesthetists" ) )  OR  ( TITLE-ABS-KEY ( anesthetists ) )  OR  ( TITLE-ABS-KEY ( anesthe* ) )  OR  ( TITLE-ABS-KEY ( anaesthe* ) ) )  AND  ( TITLE-ABS-KEY ( "google glass" ) ) | 4 | 2 |
| ( ( TITLE-ABS-KEY ( anesthesiologists ) )  OR  ( TITLE-ABS-KEY ( anesthesiology ) )  OR  ( TITLE-ABS-KEY ( "anesthesia department hospital" ) )  OR  ( TITLE-ABS-KEY ( "nurse anesthetists" ) )  OR  ( TITLE-ABS-KEY ( anesthetists ) )  OR  ( TITLE-ABS-KEY ( anesthe* ) )  OR  ( TITLE-ABS-KEY ( anaesthe* ) ) )  AND  ( TITLE-ABS-KEY ( "google glasses" ) ) | 4 | 2 |
| ( ( TITLE-ABS-KEY ( anesthesiologists ) )  OR  ( TITLE-ABS-KEY ( anesthesiology ) )  OR  ( TITLE-ABS-KEY ( "anesthesia department hospital" ) )  OR  ( TITLE-ABS-KEY ( "nurse anesthetists" ) )  OR  ( TITLE-ABS-KEY ( anesthetists ) )  OR  ( TITLE-ABS-KEY ( anesthe* ) )  OR  ( TITLE-ABS-KEY ( anaesthe* ) ) )  AND  ( TITLE-ABS-KEY ( "head up display" ) ) | 10 | 8 |
| **Searches in context “alarm management” and “vital signs” 2018-04-12** | | |
| TITLE-ABS-KEY ( "clinical alarms" ) | 711 | - |
| TITLE-ABS-KEY ( "monitoring physiologic" ) | 46,416 | - |
| TITLE-ABS-KEY ( "vital signs" ) | 20,674 | - |
| TITLE-ABS-KEY ( "medical order entry systems" ) | 2,000 | - |
| TITLE-ABS-KEY ( monitor* ) | 2,004,522 | - |
| TITLE-ABS-KEY ( alarm* ) | 87,546 | - |
| TITLE-ABS-KEY ( vital* ) | 314,121 | - |
| TITLE-ABS-KEY ( "head mounted display" ) | 3,987 | - |
| TITLE-ABS-KEY ( "hands free" ) | 2,986 | - |
| TITLE-ABS-KEY ( alert* ) | 74,624 | - |
| ( ( TITLE-ABS-KEY ( alert* ) )  OR  ( ( TITLE-ABS-KEY ( "clinical alarms" ) )  OR  ( TITLE-ABS-KEY ( "monitoring physiologic" ) )  OR  ( TITLE-ABS-KEY ( "vital signs" ) )  OR  ( TITLE-ABS-KEY ( "medical order entry systems" ) )  OR  ( TITLE-ABS-KEY ( monitor* ) )  OR  ( TITLE-ABS-KEY ( alarm* ) )  OR  ( TITLE-ABS-KEY ( vital* ) ) ) )  AND  ( ( TITLE-ABS-KEY ( eyeglasses ) )  OR  ( TITLE-ABS-KEY ( eyeglass ) )  OR  ( TITLE-ABS-KEY ( eyeglas* ) ) )  Limits 1: Filter by subject area: Medicine, Health professions, psychology, Neuroscience, Decision sciences and Nursing.  Limits 2: Excluding all articles about Ophthalmology, aviation, radiology and dentist. | \| 273 \|  \| \| --- \| --- \|   After Limits 1: 199  After limits 2:  79 | 4 |
| ( ( TITLE-ABS-KEY ( alert* ) )  OR  ( ( TITLE-ABS-KEY ( "clinical alarms" ) )  OR  ( TITLE-ABS-KEY ( "monitoring physiologic" ) )  OR  ( TITLE-ABS-KEY ( "vital signs" ) )  OR  ( TITLE-ABS-KEY ( "medical order entry systems" ) )  OR  ( TITLE-ABS-KEY ( monitor* ) )  OR  ( TITLE-ABS-KEY ( alarm* ) )  OR  ( TITLE-ABS-KEY ( vital* ) ) ) )  AND  ( TITLE-ABS-KEY ( glass ) ) | 20,886 | - |
| ( ( TITLE-ABS-KEY ( alert* ) )  OR  ( ( TITLE-ABS-KEY ( "clinical alarms" ) )  OR  ( TITLE-ABS-KEY ( "monitoring physiologic" ) )  OR  ( TITLE-ABS-KEY ( "vital signs" ) )  OR  ( TITLE-ABS-KEY ( "medical order entry systems" ) )  OR  ( TITLE-ABS-KEY ( monitor* ) )  OR  ( TITLE-ABS-KEY ( alarm* ) )  OR  ( TITLE-ABS-KEY ( vital* ) ) ) )  AND  ( TITLE-ABS-KEY ( glasses ) ) | 20,886 | - |
| ( ( TITLE-ABS-KEY ( alert* ) )  OR  ( ( TITLE-ABS-KEY ( "clinical alarms" ) )  OR  ( TITLE-ABS-KEY ( "monitoring physiologic" ) )  OR  ( TITLE-ABS-KEY ( "vital signs" ) )  OR  ( TITLE-ABS-KEY ( "medical order entry systems" ) )  OR  ( TITLE-ABS-KEY ( monitor* ) )  OR  ( TITLE-ABS-KEY ( alarm* ) )  OR  ( TITLE-ABS-KEY ( vital* ) ) ) )  AND  ( TITLE-ABS-KEY ( "smart glass" ) ) | 63 | 6 |
| ( ( TITLE-ABS-KEY ( alert* ) )  OR  ( ( TITLE-ABS-KEY ( "clinical alarms" ) )  OR  ( TITLE-ABS-KEY ( "monitoring physiologic" ) )  OR  ( TITLE-ABS-KEY ( "vital signs" ) )  OR  ( TITLE-ABS-KEY ( "medical order entry systems" ) )  OR  ( TITLE-ABS-KEY ( monitor* ) )  OR  ( TITLE-ABS-KEY ( alarm* ) )  OR  ( TITLE-ABS-KEY ( vital* ) ) ) )  AND  ( TITLE-ABS-KEY ( "smart glasses" ) ) | 63 | 6 |
| ( ( TITLE-ABS-KEY ( alert* ) )  OR  ( ( TITLE-ABS-KEY ( "clinical alarms" ) )  OR  ( TITLE-ABS-KEY ( "monitoring physiologic" ) )  OR  ( TITLE-ABS-KEY ( "vital signs" ) )  OR  ( TITLE-ABS-KEY ( "medical order entry systems" ) )  OR  ( TITLE-ABS-KEY ( monitor* ) )  OR  ( TITLE-ABS-KEY ( alarm* ) )  OR  ( TITLE-ABS-KEY ( vital* ) ) ) )  AND  ( TITLE-ABS-KEY ( "google glass" ) ) | 72 | 9 |
| ( ( TITLE-ABS-KEY ( alert* ) )  OR  ( ( TITLE-ABS-KEY ( "clinical alarms" ) )  OR  ( TITLE-ABS-KEY ( "monitoring physiologic" ) )  OR  ( TITLE-ABS-KEY ( "vital signs" ) )  OR  ( TITLE-ABS-KEY ( "medical order entry systems" ) )  OR  ( TITLE-ABS-KEY ( monitor* ) )  OR  ( TITLE-ABS-KEY ( alarm* ) )  OR  ( TITLE-ABS-KEY ( vital* ) ) ) )  AND  ( TITLE-ABS-KEY ( "google glasses" ) ) | 72 | 9 |
| ( ( TITLE-ABS-KEY ( alert* ) )  OR  ( ( TITLE-ABS-KEY ( "clinical alarms" ) )  OR  ( TITLE-ABS-KEY ( "monitoring physiologic" ) )  OR  ( TITLE-ABS-KEY ( "vital signs" ) )  OR  ( TITLE-ABS-KEY ( "medical order entry systems" ) )  OR  ( TITLE-ABS-KEY ( monitor* ) )  OR  ( TITLE-ABS-KEY ( alarm* ) )  OR  ( TITLE-ABS-KEY ( vital* ) ) ) )  AND  ( TITLE-ABS-KEY ( "head up display" ) )  Limits: Filter by subject area: Medicine, Health professions, psychology, Neuroscience and Decision sciences. | \| 189 \|  \| \| --- \| --- \|   After Limits:  37 | 5 |
| ( ( TITLE-ABS-KEY ( alert* ) )  OR  ( ( TITLE-ABS-KEY ( "clinical alarms" ) )  OR  ( TITLE-ABS-KEY ( "monitoring physiologic" ) )  OR  ( TITLE-ABS-KEY ( "vital signs" ) )  OR  ( TITLE-ABS-KEY ( "medical order entry systems" ) )  OR  ( TITLE-ABS-KEY ( monitor* ) )  OR  ( TITLE-ABS-KEY ( alarm* ) )  OR  ( TITLE-ABS-KEY ( vital* ) ) ) )  AND  ( TITLE-ABS-KEY ( "head mounted display" ) )  Limits: Filter by subject area: Medicine, Health professions, psychology, Neuroscience, Decision sciences, Nursing and multidiciplinary | 343  After  Limits:  100 | 10 |
| ( ( TITLE-ABS-KEY ( alert* ) )  OR  ( ( TITLE-ABS-KEY ( "clinical alarms" ) )  OR  ( TITLE-ABS-KEY ( "monitoring physiologic" ) )  OR  ( TITLE-ABS-KEY ( "vital signs" ) )  OR  ( TITLE-ABS-KEY ( "medical order entry systems" ) )  OR  ( TITLE-ABS-KEY ( monitor* ) )  OR  ( TITLE-ABS-KEY ( alarm* ) )  OR  ( TITLE-ABS-KEY ( vital* ) ) ) )  AND  ( TITLE-ABS-KEY ( "hands free" ) )  Limits: Filter by subject area: Medicine, Health professions, psychology, Neuroscience, Decision sciences, Nursing and multidiciplinary | 206  After Limits:  69 | 4 |
| **Searches in context ”patient safety” 2018-04-10** | | |
| TITLE-ABS-KEY ( "patient safety" ) | 103,190 | - |
| ( TITLE-ABS-KEY ( "patient safety" ) )  AND  ( ( TITLE-ABS-KEY ( eyeglasses ) )  OR  ( TITLE-ABS-KEY ( eyeglass ) )  OR  ( TITLE-ABS-KEY ( eyeglas* ) ) ) | 19 | 1 |
| ( TITLE-ABS-KEY ( "patient safety" ) )  AND  ( TITLE-ABS-KEY ( glass ) ) | 189 | - |
| ( TITLE-ABS-KEY ( "patient safety" ) )  AND  ( TITLE-ABS-KEY ( glasses ) ) | 189 | - |
| ( TITLE-ABS-KEY ( "patient safety" ) )  AND  ( TITLE-ABS-KEY ( "smart glass" ) ) | 2 | 1 |
| ( TITLE-ABS-KEY ( "patient safety" ) )  AND  ( TITLE-ABS-KEY ( "smart glasses" ) ) | 2 | 1 |
| ( TITLE-ABS-KEY ( "patient safety" ) )  AND  ( TITLE-ABS-KEY ( "google glass" ) ) | 6 | 1 |
| ( TITLE-ABS-KEY ( "patient safety" ) )  AND  ( TITLE-ABS-KEY ( "google glasses" ) ) | 6 | 1 |
| ( TITLE-ABS-KEY ( "patient safety" ) )  AND  ( TITLE-ABS-KEY ( "head up display" ) ) | 5 | 0 |
