# Supplementary material for: Smart Glasses for Caring Situations in Complex Care Environments: Scoping Review
Source: JMIR Mhealth Uhealth. 2020 Apr 20;8(4):e16055. doi: 10.2196/16055 (PMC7199139; doi:10.2196/16055)
Supplement: Multimedia Appendix 3 [file mhealth_v8i4e16055_app3.docx]

## Appendix 3: Charting of articles included in the qualitative content analysis

| Author, no in reference list, year, country^a^, type^b^ | Title | Aim of the study | Design, method, context and participants | Important findings |
| --- | --- | --- | --- | --- |
| Albrecht, U-V., von Jan, U., Kuebler, J., Zoeller, C., Lacher, M., Muensterer, O. J., Ettinger, M., Klintschar, M. & Hagemeier, L.  No 36  2014  Germany  Original paper | Google Glass for Documentation of Medical Findings: Evaluation in forensic Medicine | To empirically determine the feasibility of deploying Glass in a forensics setting. | 2 physicians used glasses to document autopsy and post-mortem examination of 4 decedents. Afterwards interviews were made.  159 pictures taken by glasses n=86 (5-mpix) and digital single-lens reflex (DSLR) camera n=73 (12,6 mpix), were compared by 6 forensic examiners (including the 2 physicians) to evaluate image quality. | Glass were sufficient for documentation in forensic medicine, but image quality was inferior compared to DSLR camera. Glasses were comfortable to wear. Effort necessary to use glasses were higher and examinations took longer time. Battery capacity in glasses is one disadvantage, and further development is needed for professional use. |
| Aldaz, G., Shluzaz, L. A., Pickham, D., Eris, O., Sadler, J., Joshi, S. & Leifer, L.  No 37  2015  USA  Research article | Hands-Free Image Capture, Data Tagging and Transfer Using Google Glass: A Pilot Study for Improved Wound Care Management | To present the design and lab-based assessment of the Snap-Cap System, a Google Glass and Android smartphone-based application capable of mobile, hands-free image capture. | Three parts of a project is described.  Development of a hands-free image capture system (Snap-Cap) based on needs found during interviews with physicians and nurses, and observations of nurses. Feasibility and usage assessment were made with 7 participants (physicians and nurses).  A pilot study with 16 wound care nurses in lab environment. They took pictures using the new system (see above), and existing photo documentation system, and compared them using a questionnaire.  Follow-up evaluation on speech-to-text for wound annotation using Word Error Rate. | Nurses preferred Snap-Cap to identify patients through barcode scanning, using voice commands and possibility of sterile image-capturing. Use of head gestures to control glasses needed improvement. Opinion about image quality and overall ease of use were divided.  Speech-to-text were encouraging but needs extended specified vocabulary related to context. |
| Drake-Bockman, T. F. E., Datta, A. & von Ungern-Sternberg, B. S.  No 23  2016  Australia  Research report | Patient monitoring with Google Glass: a pilot study of a novel monitoring technology | To examine the acceptance of Google Glass as a patient monitoring device in a pediatric anesthesia context at Princess Margaret Hospital for Children, Perth, Australia. | 40 anesthetists with different levels of experience were included in a pilot study and used Google Glasses for patient monitoring for the duration of a theater list in pediatric surgery. They handled 1-4 cases each. Data was recorded during the day and participants answered a questionnaire after participating. | 90% found Glasses comfortable, 86% found it easy to read, 82,5% found it non-distracting. 75% were comfortable using Glasses in theater, 76% would use it again and 58% would recommend it to a colleague. |
| Drummond, D., Arnaud, C., Guedj, R., Duguet, A., de Suremain, N. & Petit, A.  No 34  2017  France | Google Glass for Residents Dealing With Pediatric Cardiopulmonary Arrest: A Randomized, Controlled, Simulation-Based Study | To determine whether real-time video communication between the first responder and a remote intensivist via Google Glass improves the management of a simulated in-hospital pediatric cardiopulmonary arrest before the arrival of the ICU team. | Randomized controlled study where 42 first-year residents in pediatrics performed pediatric CPR on manikin, 2 cases each. The intervention group wore Google Glasses. Glasses could be used to seek help from remote intensivist using real-time video in the second case. | Real-time video communication with intensivist did not affect no-blow or no-flow fractions. Quality of insufflations and chest compressions were improved using real-time video communication through Google Glass. |
| Fernando, S., Wang, W., Kirenko, I., de Haan, G., Bambang-Oetomo, S. & Corporaal, H.  No 27  2015  Netherlands  Conference article | Feasibility of Contactless Pulse Rate Monitoring of Neonates using Google Glass | To investigate the feasibility of: (1) monitoring the pulse rate of neonates in a clinical setting with a camera-based algorithm and (2) using Google Glass with such an algorithm. | Information about 7 neonates’ pulse rate (during 1-32 min) from a new algorithm were presented on Google Glasses and compared to reference pulse data. | The Google Glass concept was feasible for wearable pulse rate monitoring. |
| Gupta, S., Boehme, J., Manser, K., Dewar, J., Miller, A., Siddiqui, G. & Schwaitzberg, S. D.  No 32  2016  USA | Does Wearable Medical Technology With Video Recording Capability Add Value to On-Call Surgical Evaluations? | Explore the use and potential value of an asynchronous, near-real time protocol – which avoids transmission issues associated with real-time applications – for recording, uploading, and viewing of HD visual media with Google Glass in the ED to facilitate remote surgical consults.  Explore surgeon opinion about use of Google Glass during consultation and patient perception about providers wearing medical technology with recording capability. | 7 surgical attendings on home-call and 4 physician assistants used Google Glass to capture 45 physical examination and diagnostics of patients with surgical complaints in an ED. Afterwards surgical consultation was made by telephone as conventional, and then by assessing the captured video, and the advice from consultations were compared.  Attending surgeons answered a survey about their opinions of use.  276 patients answered a survey about their opinion on smart glasses included in their care. | The management plans changed in 24% of the cases after assessing the captured video.  Watching the video made the consultants feel more confident in their management plan in 44,4%, no change in 44,4% and decreased confidence in 11,1%.  Patients were comfortable with the use of wearable technology in their care. |
| Garcia-Cruz, E., Bretonnet, A. & Alcaraz, A.  No 31  2018  Spain | Testing Smart Glasses in urology: Clinical and Surgical potential application | To explore the potential benefits of using smart glasses – wearable computer optical devices with touch-less command features – in the surgery room and in outpatient care settings in urology. | 80 urologists and their colleagues (a total of 240) used smart glasses during 7 months in daily clinical and surgical practice. The initial 80 participants rated usefulness of smart glasses on a 10-point scale during telephone interviews using a semi-structured questionnaire, where also insights on potential benefits were provided. | Glasses were found to be more useful in the surgery room then in outpatient clinics. In surgery setting live streaming video, images, education and consulting were found most useful. In outpatient clinics glasses were found useful for viewing test results, drug prescriptions and medical records, particularly in emergency situations. |
| Iqbal, M. H., Aydin, A., Lowdon, A., Ahmed, H. I., Muir, G. H., Khan., M. S., Dasgupta, P. & Ahmed, K.  No 28  2016  United Kingdom  Original research | The effectiveness of Google Glass as vital signs monitor in surgery: A simulation study | To determine whether the Google Glass increases the awareness of patient vital signs and whether such devices are intrusive or detrimental to the surgeon’s direct or peripheral vision and technical performance. | 24 medical students and 13 urologists at different levels performed a procedure in simulated setting using standard vital signs monitor and then Google Glass. Time to respond to abnormal vital signs during both sessions were recorded. Objective outcome was measured. User feedback were collected by survey. | A significant majority responded quicker to abnormal vital signs using Google Glass. There were no significant differences in technical performance using or not using Glasses. A significant majority agreed that Glass increased their awareness of vital signs and would like to use them again. |
| Jeroudi, O. M., Christakopoulos, G., Christopoulos, G., Kotsia, A, Kypreos, M. A., Rangan, B. V., Banerjee, S. & Brilakis, E. S.  No 40  2015  USA | Accuracy of Remote Electrocardiogram Interpretation With the Use of Google Glass Technology | To evaluate the use of Google Glass for remote electrocardiographic interpretation. | 12 cardiologists interpreted images of 10 ECGs from a book in 4 different ways: images captured by the camera of Google Glass viewed in Google Glass, on mobile phone and on paper, and high resolution image of ECG on mobile phone. Accuracy of interpretation and user experiences were measured. | Interpretation of ECG viewed in Glass was less accurate than viewed on paper. The same image of ECG was more accurate interpreted on a mobile phone than in Glass, but viewed on paper gave the most accurate result.  75% of the cardiologists was dissatisfied with ECG-viewing on Google Glass prism and 83% were not confident in their interpretation. |
| Kassutto, S. M., Kayser, J. B., Kerlin, M. P, Upton, M., Lipschik, G., Epstein, A. J., Dine, C. J. & Schweickert, W.  No 35  2017  USA | Google Glass Video Capture of Cardiopulmonary Resuscitation Events: A Pilot Simulation Study | To understand the feasibility of Google Glass as a method for recording inpatient cardiac arrests and capturing salient resuscitation factors for post-event review. | An observational study where 11 simulated cardiac arrests in inpatient setting were reviewed by 3 methods; in-room direct observations, stationary video camera and video from Google Glass worn by physician code leader. Nurse and physician specialists analysed the videos afterwards. Code leaders were surveyed about Google Glasses (n=10). | Google Glasses performed slightly better than stationary video camera regarding visualization and audibility. Survey responders agreed that glasses were easy to use, but 2 found them distracting. All 10 responders wanted more feedback on their performance during clinical emergencies, but 3 would be uncomfortable using them during actual resuscitation. |
| Liebert, C. A., Zayed, M. A., Aalami, O., Tran, J. & Lau, J. N.  No 29  2016  USA | Novel Use of Google Glass for Procedural Wireless Vital Sign Monitoring | To investigate the feasibility and potential utility of head-mounted displays for real-time wireless vital sign monitoring during surgical procedures. | 14 surgery residents participated in a randomized controlled pilot study performing bedside bronchoscopy and thoracostomy tube placement on mannequin. 7 wore Google Glasses as addition to traditional monitor for vital sign monitoring for each of the two cases. Objective outcome was measured. User feedback were collected by survey. | The Google Glass group spent significantly less time looking away from the procedural field to view monitor. The trend showed earlier recognition of deterioration of vital sign, but this was not significant. The majority felt that Google Glass increased situational awareness, was helpful in monitoring vitals, was easy to use and could improve patient safety. |
| Muensterer, O. J., Lacher, M., Zoeller, C., Bronstein, M. & Kübler, J.  No 22  2014  USA  Original research | Google Glass in pediatric surgery: An exploratory study | To test Google Glass applicability in our daily pediatric surgical practice and in relevant experimental settings. | Google Glass was worn by one of the authors (medical doctor) during 4 weeks of clinical practice in all potential situations. | Google Glass were used in several situations. Clear utilities, such as handsfree photo- and video documentation, handsfree phone calls and internet searches, were seen in clinical setting. Improvements of hardware are suggested as well as specialized medical applications and improvement in data protection. |
| Schaer, R., Salamin, F., Jiménez del Toro, O. A., Müller, H. & Widmer, A.  No 41  2015  Switzerland  Conference article | Live ECG Readings using Google Glass in Emergency Situations | To compare 6 ECG rhythms readings from a 13,3 inch laptop screen and from the prism of Google Glass. | An experimental study involving 7 medical residents in internal medicine with experience of emergency department. 4 patient vital signs (ECG, pulse, blood pressure and oxygen saturation) for 6 simulated patients were shown in Google Glasses and on a laptop screen. The 6 patients were given different ECG rhythms that needed urgent attention. Time to recognition were measured. | No significant difference was found between the two testing conditions. Some fatigue was reported after reading ECG on Google Glass. |
| Skolnik, A. B., Chai, P. R., Dameff, C., Gerkin, R., Monas. J., Padilla-Jones, A. & Curry, S.  No 33  2016  USA  Original article | Teletoxicology: Patient Assessment Using Wearable Audiovisual Streaming Technology | To evaluate the quality of the poisoned patient assessment via wearable audiovisual streaming technology. | In a prospective observational cohort study 50 poisoned patient assessments were filmed by on-site investigators with Google Glass and transmitted to a remote investigator. Photographs of ECG were taken and transmitted. Examinations were made separately and compared. A survey about acceptability and reliability were performed. | Remote evaluation of poisoned patients was similar when it came to examination findings and ECG interpretation. Interpretation of pupil size and response to light had lowest agreement.  High degree of comfort using Google Glass were reported. Remote investigators perceived reliability lower than on-site investigators. |
| Spaedy, E., Christakopoulos, G. E., Tarar, M. N. J., Christopoulos, G., Rangan, B. V., Roesle, M., Ochoa, C. D., Yarbrough, W., Banerjee, S. & Brilakis, E. S.  No 42  2016  USA  Original research | Accuracy of remote chest X-ray interpretation using Google Glass technology | To explore the accuracy of remote chest X-ray reading using hands-free, wearable technology. | 15 physicians interpreted 12 chest X-rays each, presented in 3 different ways: in Google Glass prism, Google Glass photograph of X-ray presented on mobile phone and original X-ray presented on desktop computer. Accuracy of interpretation was measured, as well as user experience. | Chest X-ray interpretation was less accurate using Google Glass than interpretation using desktop computer or mobile phone. 87% of participants were dissatisfied with image of chest X-ray in Google Glass. Participants saw potential to use of Google Glass for other medical purposes. |
| Spencer. R. J., Chang, P. H., Guimaraes, A. R. & Firth, P. G.  No 38  2014  USA  Case report | The use of Google Glass for airway assessment and management | Explore whether Google Glass has application in airway management. | 2 cases of real tracheal intubation were captured with Google Glass. | Google glass can be used to document airway assessment and management in standard lightning with minimal disruption to workflow. |
| Stetler, J., Resendes, E., Martinez-Parachini, J. R., Patel, K., Amsavelu, S., Tarar, M. N. J., Christakopoulus, G. E., Rangan, B. V., Rosele, M., Abdullah, S., Obel, O., Grodin, J., Banerjee, S. & Brilakis, E.  No 43  2016  USA  Correspondence | Hands-free zoom and pan technology improves the accuracy of remote electrocardiogram interpretation using Google Glass | To use a novel software for zoom and pan on Google Glass for ECG interpretation. | 12 cardiologists interpreted 10 ECGs on 3 different platforms: viewed in Google Glass, on mobile phone and on paper. Glass and mobile phone could zoom and pan. ECGs interpreted were images of ECGs from a book captured by the camera of Google Glass. Accuracy of interpretation and user experiences were measured. | Interpretation of ECGs on Google Glass with zoom and pan software was non-inferior to interpretation on paper and on a mobile phone. Physician satisfaction with Google Glass was high. About half of the physicians was satisfied with image quality and confident in their interpretation. |
| Udani, A. D., Harrison, T. K., Howard, S. K., Kim, T. E., Brock-Utne, J. G., Gaba, D. M. & Mariano, E. R.  No 44  2012  USA | Preliminary Study of Ergonomic Behaviour During Simulated Ultrasound-Guided Regional Anesthesia Using a Head-Mounted Display | To evaluate the feasibility of using head-mounted display technology to improve ergonomics in ultrasound-guided regional anesthesia in a simulated environment. | In a pilot study 2 anaesthesiologists performed 5 ultrasound-guided nerve blocks each on a pig using a head mounted display for viewing ultrasound image. An observer assessed ergonomics and overall block quality. User experience were asked for. | All procedures were adequately completed. Neither practitioner showed poor ergonomic behaviour, specifically neither practitioner redirected their attention away from the procedural field. |
| Wu, T. S., Dameff, C. J. & Tully, J. L.  No 45  2014  USA | Ultrasound-guided central venous access using Google Glass | To evaluate whether or not medical practitioners at various levels of training could use Google Glass to perform an ultrasound-guided procedure, and to explore potential advantages of this technology. | In a pilot study 40 participants (emergency medicine residents and medical students) with different levels of training participated. 20 used Google Glass when performing ultrasound-guided central line on mannequin, 20 used traditional ultrasound guidance. Task were performed 2 times each. Performance were measured, and user experience gathered in post-exercise survey. | All participants managed to complete the task. Google Glass wearers took longer time to gain access, and had more needle redirections, but less head movements were noted. |
| Wüller, H., Behrens, J., Klinker, K., Wiesche, M., Krcmar, H. & Remmers, H.  No 39  2018  Germany  Conference article | Smart Glasses in Nursing – Situation Change and Further Usages Exemplified on a Wound Care Application | How does the usage of smart glass applications change the situation of care giving?  Which ideas for future usage of smart glasses in nursing do nurses have? | Through information from workshops and interviews an application for wound care were developed and implemented on Wuzix glasses. 5 nurses used the application for a wound care documentation in nursing home context and semi-structured interviews were made afterwards. Interviews were analysed using qualitative content analysis. | Smart glasses for wound care management may change the situation in many ways. Documentation might be enhanced, but communication with the patient were challenged. Nurses opinion about smart glasses in nursing varied. |

^a^In articles where no country for where the study took place is specified, the authors country is stated as country.

^b^Article type is not written out if not stated in the article.
